# Integrated Single-Cell Analysis Revealed Novel Subpopulations of Foamy Macrophages in Human Atherosclerotic Plaques

**DOI:** 10.3390/biom14121606

**Published:** 2024-12-16

**Authors:** Yunrui Lu, Shuang Wu, Shiyu Zhu, Jian Shen, Chang Liu, Chaoyue Zhao, Sheng’an Su, Hong Ma, Meixiang Xiang, Yao Xie

**Affiliations:** State Key Laboratory of Transvascular Implantation Devices, Heart Regeneration and Repair Key Laboratory of Zhejiang Province, Department of Cardiology, The Second Affiliated Hospital, Zhejiang University School of Medicine, Hangzhou 310009, China; luyunrui@zju.edu.cn (Y.L.); 12318458@zju.edu.cn (S.W.); 3160103655@zju.edu.cn (S.Z.); shenjian27753@zju.edu.cn (J.S.); liuc@zju.edu.cn (C.L.); 22218012@zju.edu.cn (C.Z.); chearbear@zju.edu.cn (S.S.); hong_ma@zju.edu.cn (H.M.)

**Keywords:** atherosclerosis, foam cells, macrophages, integrated single-cell analysis, single-cell RNA sequencing, Mendelian randomization

## Abstract

Foam cell formation is a hallmark of atherosclerosis, yet the cellular complexity within foam cells in human plaques remains unexplored. Here, we integrate published single-cell RNA-sequencing, spatial transcriptomic, and chromatin accessibility sequencing datasets of human atherosclerotic lesions across eight distinct studies. Through this large-scale integration of patient-derived information, we identified foamy macrophages enriched for genes characteristic of the foamy signature. We further re-clustered the foamy macrophages into five unique subsets with distinct potential functions: (i) pro-foamy macrophages, exhibiting relatively high inflammatory and adhesive properties; (ii) phagocytic foamy macrophages, specialized in efferocytosis; (iii) high-efflux foamy macrophages marked by high *NR1H3* expression; (iv) mature foamy macrophages prone to programmed cell death; and (v) synthetic subset. Trajectory analysis elucidated a bifurcated differentiation cell fate from pro-foam macrophages toward either the programmed death (iv) or synthetic (v) phenotype. The existence of these foamy macrophage subsets was validated by immunostaining. Moreover, these foamy macrophage subsets exhibited strong potential ligand–receptor interactions. Finally, we conducted Mendelian randomization analyses to identify a possible causal relationship between key regulatory genes along the programmed death pathway in foamy macrophages and atherosclerotic diseases. This study provides a high-resolution map of foam cell diversity and a set of potential key regulatory genes in atherosclerotic plaques, offering novel insights into the multifaceted pathophysiology underlying human atherosclerosis.

## 1. Introduction

Atherosclerosis is a chronic inflammatory disease driven by impaired lipid metabolism [1] and is a leading cause of cardiovascular diseases [2]. Foam cells, characterized by intracellular accumulation of lipid molecules, comprise the major cellular component of atherosclerotic lesions and play critical roles in the initiation and progression of atherosclerotic plaques [3].

In the course of hyperlipidemia and atherosclerosis, macrophages are recruited to the intima, where they uptake excessive lipoproteins via scavenger receptors (SRs), transforming into cholesterol-enriched foam cells. These cells exhibit increased expression of lipid-processing genes and decreased inflammatory gene expression [4,5]. Dysfunction in cholesterol efflux, as well as the excessive lipid metabolism in foamy macrophages, causes macrophage death, contributing to necrotic core formation in atherosclerotic plaques [6]. Foam cells, especially macrophage foam cells, which are induced by hyperlipidemia, directly dictate lesion progression or regression by affecting atherosclerotic stability through cellular responses, such as cytokine secretion, apoptosis, necrosis, and efferocytosis [4,7]. The complexity of cellular responses suggests the cellular heterogeneity of foamy macrophages. Unraveling the diverse subtypes of foamy macrophages is crucial for deciphering the intricate tapestry of atherosclerotic pathophysiology.

Although atherosclerotic lesions exhibit site-specific characteristics, major features of atherosclerotic pathogenesis are overall conserved across vascular regions. Thanks to the advancement of single-cell RNA sequencing (scRNA-seq) and single-cell ATAC sequencing (scATAC-seq), the full immune and non-immune cell composition of murine and human atherosclerotic plaques has been described recently [5,8,9,10,11]. *TREM2*^hi^ macrophages were identified with specialized functions in lipid metabolism and catabolism [12]. A population of *TREM2*-expressing foam cell-like macrophages with a fibrosis-promoting phenotype has been reported in human carotid plaques [8]. Meta-analysis of murine atherosclerotic plaques identified two *Trem2^hi^* clusters (*Trem2^hi^-Slamf9* and *Trem2^hi^-Gpnmb*) enriched for foamy/*Trem2^hi^* signature genes [9]. However, these two subpopulations were not found in human plaque analysis, probably due to the limited cell number [9]. Recent research characterizing human carotid atherosclerosis identified 2 *TREM2*^+^ foam cell clusters, one expressing *ABCA1*, *LPL*, and *CD36*, and another expressing *APOE* along with inflammatory genes like *CCL18* and *C1Q* [13]. While current sequencing studies have successfully clustered macrophages from atherosclerotic plaques and identified macrophage subpopulations, including foam-like macrophages, the detailed cellular heterogeneity, as well as the potential cell-fate and cell-cell communication within foam macrophages at a higher resolution, remain uncharted in human plaques.

Here, we integrate single-cell RNA-sequencing, spatial transcriptomic, and chromatin accessibility sequencing datasets of human atherosclerotic plaques associated with Mendelian randomization (MR) analyses. We observed five different subsets with distinct putative functions in foamy macrophages, the existence of which was confirmed by human sample staining. Functional enrichment, pseudotime trajectory, TF regulon activity, cell–cell communication analyses, and spatial prediction further provided a higher-resolution view of the relationship within foam subpopulations. MR analysis revealed the causal relationship between foamy macrophage programmed cell death genes and the risk of atherosclerosis and myocardial infarction (MI). These insights into the landscape and biological features of foam populations offer valuable information for atherosclerotic interventions by targeting foam cells.

## 2. Materials and Methods

### 2.1. Single-Cell Data Integration

**Figure 1:**
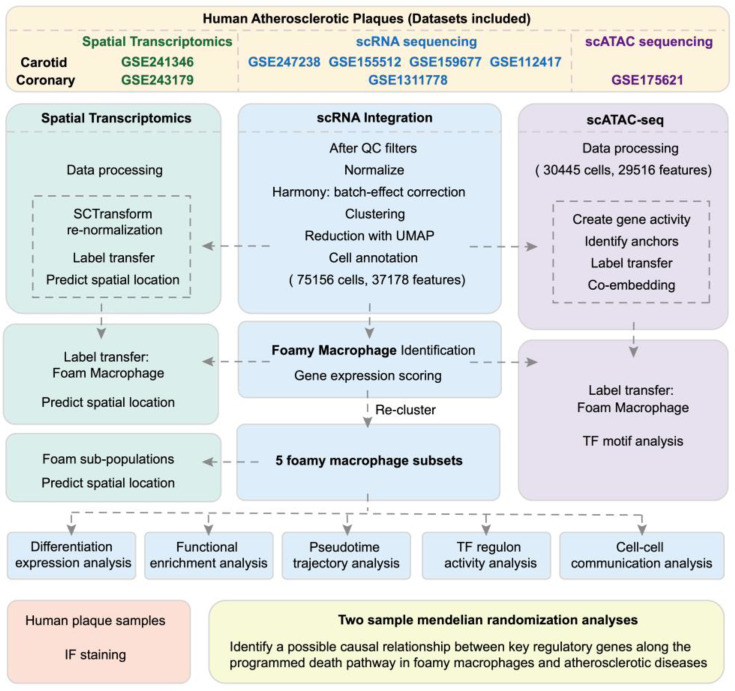
Pipeline devised to build the integrated single-cell analyses for human atherosclerosis.

Using 37 public distinct human scRNA-seq libraries from 25 patients across 7 studies, coupled with 44 public distinct human scATAC-seq datasets from 41 patients in 1 study, we performed integrated analyses (Figure 1 and Figure S1). All of these public datasets adhered to the Declaration of Helsinki. The analytical workflow comprised three key steps: (i) integrating all scRNA-seq data, (ii) integrating scATAC-seq data with scRNA-seq data, and (iii) integrating spatial datasets with scRNA-seq data. The integrated data comprised the atherosclerotic lesion (group ‘Plaque’) and non-lesion artery (group ‘Control’). ‘Control’ samples of carotid arteries were proximal adjacent portions of carotid artery tissue harvested during carotid endarterectomy or open repair surgery. ‘Control’ samples of coronary arteries were from heart transplant recipients or rejected donor hearts and without atherosclerotic lesions. Deposited cell–gene count matrixes were analyzed in *Seurat*. All datasets were pre-processed for quality control (Figure S2A) and metadata information (Table S1). Cells with unique feature counts > 7000 or <250 or with >20% mitochondrial counts or >3% hemoglobin counts were filtered out. For nCounts, the bottom 3% of the data were removed, and only cells with UMI counts > 500 were selected. *Harmony* 1.2.0 package was used to integrate scRNA-seq data across multiple batches. *Seurat’s* ‘Anchor’-based integration workflow was applied in scATAC-seq integration and spatial data integration. Further analysis of scATAC-seq was conducted using *Signac* 1.12.0.

### 2.2. Single-Cell Data Analyses

#### 2.2.1. Doublet Removal

Doublet prediction was performed using the *DoubletFinder* 2.0.4 package, which identifies doublets formed from transcriptionally distinct cells. Moreover, cells of one cluster co-expressing discrepant lineage markers were further removed.

#### 2.2.2. Gene Set Enrichment Analysis

Functional enrichment analysis was performed using *ClusterProfiler* 4.10.0 with a list of differential expression genes. Each cluster was analyzed for Kyoto Encyclopedia of Genes and Genomes (KEGG) pathways and Gene Ontology (GO) with an adjusted *p*-value < 0.05 used to identify the significantly enriched gene sets. *AUCell* 1.24.0 was conducted to identify cells with active gene sets, presenting the enrichment of a specific pathway within clustered cells.

#### 2.2.3. Cell Trajectory Analysis

*Monocle3* 1.3.5 was instrumental in constructing the single-cell pseudotime trajectory. Manual annotations were overlaid on the UMAP. The spots predominantly comprising foam cells in the early stage of atherosclerosis (group ‘Control’), characterized by high enrichment of the inflammatory pathway but low enrichment of cholesterol metabolism, were marked as “roots” of the trajectory. Order_cells() function was called to calculate where each cell falls in pseudotime.

#### 2.2.4. Cell–Cell Communication Analysis

*CellChat* 1.6.1 facilitated the inference, analysis, and visualization of cell–cell communication from single-cell data. The major signaling inputs and outputs for all cell populations and ligand–receptor interactions were predicted by *CellChat* 1.6.1.

#### 2.2.5. Protein–Protein Interactions Analysis

Protein–protein interactions analysis was performed using *STRINGdb* 2.14.0 with the STRING protein–protein interactions database (https://string-db.org (accessed on 21 March 2024)).

#### 2.2.6. Transcription Factors Enrichment Analysis

Transcription factors (TFs) enrichment analysis of scRNA-seq data was deciphered using *SCENIC* 1.3.1, which was performed based on the recommended pipelines (https://scenic.aertslab.org/ (accessed on 25 June 2024)).

#### 2.2.7. Data Visualization

*Ggplot2* 3.4.4, *forestploter* 1.1.1, and *scCustomize* 2.0.1 were used to optimize data visualization. These data analyses were performed using custom R Scripts (R version 4.3.2) designed especially for this research or based on the recommended pipelines from the preexisting packages listed above.

### 2.3. Foam Cell Identification

**Figure 2:**
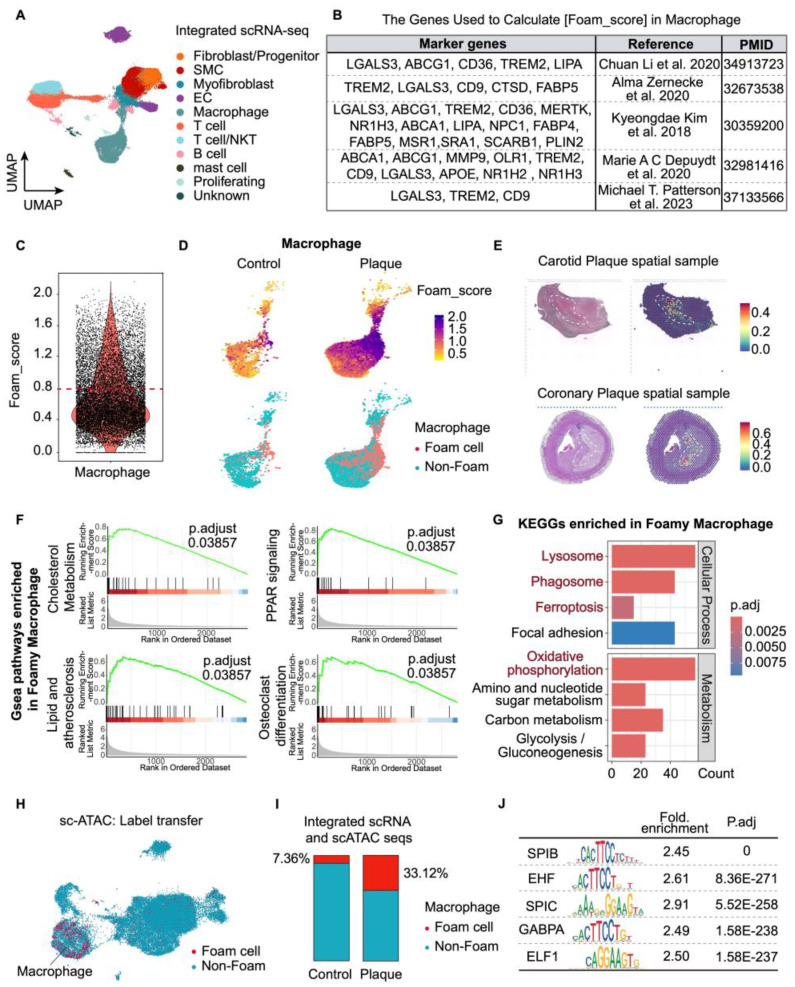
Identification and characterization of foam macrophages in human atherosclerotic plaques. (**A**) UMAP representation of integrated scRNA-seq data (75,156 cells) from human atherosclerotic plaques with identification of the major cell lineages. (**B**) Marker genes of foam cells reported in previous studies [5,8,14,15,16]. (**C**) Violin plot showing the distribution of Foam_score in scRNA-seq data. (**D**) UMAP embeddings of Foam_score and ‘Foam cell’ cluster. The Foam_score was calculated for each cell based on the expression of foam marker genes listed in (**B**). ‘Foam’ cluster was identified based on the Foam_score. (**E**) Predicted spatial location of identified foam cells in the sections of human carotid and coronary atherosclerotic plaque samples. (**F**) Top GSEA pathways associated with ‘Foam’ macrophages. (**G**) Top KEGG pathways associated with ‘Foam’ macrophages. (**H**) Projection of scRNA-seq ‘Foam cell’ and ‘Non-Foam’ labels over the scATAC-seq clusters (30,445 cells). (**I**) Bar plot showing the distribution of ‘Foam cell’ and ‘Non-Foam’ clusters across control and plaque groups in the integrated data. (**J**) Top differential open chromatin TF motifs by chromVAR in ‘Foam’ cluster. P. adj, adjusted *p*-value by Benjamini–Hochberg procedure.

The [Foam_score] was calculated according to the average expression of a set of genes related to foam cell formation (Figure 2B and Figure S4), which have been reported as differentially expressed between foam and non-foam cells by RNA sequencing. These genes were associated with lipid formation, transportation, or storage. A threshold of 0.8 was chosen to define foam cells to minimize background interference (Figure 2C). Data analyses were performed using custom R Scripts (R version 4.3.2).

### 2.4. Mendelian Randomization Analyses

MR analysis was conducted by *TwoSampleMR* 0.5.7. Tissue-specific cis-expression quantitative trait loci (cis-eQTL) summary statistics data and outcome GWAS summary data are detailed in Supplementary Table S2. Single nucleotide polymorphisms (SNPs) were selected as instrumental variants based on the following criteria: (1) SNPs associated with any protein at a genome-wide significance level (*p* < 5 × 10^−8^); (2) the 1000 Genomes Project European sample data was used as a reference panel to calculate LD among SNPs (r^2^ < 0.001); (3) exclusion of SNPs with missing data or no suitable proxies. In cases where only one IV was available, the Wald ratio was used to estimate the log odds changes in disease risk per standard deviation incremental of gene expression level. With two or more SNPs, the inverse-variance weighted method with random effect was utilized to estimate effects. Robustness was assessed using weighted mode, weighted median, simple mode, and MR-Egger. The Benjamini–Hochberg false discovery rate (FDR) method was used for multiple testing correction, with FDR < 0.05 as the significance level.

### 2.5. Immunofluorescence Staining of Human Samples

The collection of human carotid artery samples for immunofluorescence analyses was approved by the Human Research Ethics Committee of the Second Affiliated Hospital of Zhejiang University School of Medicine (Approval No. 2023LSYD0751), Hangzhou, China. Informed written consent was obtained from all study participants. Metadata from human samples used in the single-cell meta-analysis can be found in Table S1.

Human atherosclerotic plaques were collected during carotid thromboendarterectomy procedures at the vascular surgery department, Second Affiliated Hospital of Zhejiang University School of Medicine, after approval from the local ethics committees and in agreement with the Declaration of Helsinki. Informed written consent was obtained from all patients who had either symptomatic carotid stenosis or very tight asymptomatic stenosis. Carotid atherosclerotic plaques were immediately snap-frozen in liquid nitrogen and subsequently cross-sectioned at 6-µm thickness. For immunofluorescent staining, sections were fixed with 4% paraformaldehyde, then permeabilized with 0.1% Triton X-100, and blocked with 50% donkey serum in PBS. Sections were incubated with primary antibodies overnight at 4 °C and then stained by secondary antibodies for 1 h at room temperature.

## 3. Results

### 3.1. Integrated Single-Cell Analyses of Human Atherosclerotic Plaques

By integrating scRNA-seq data of human atherosclerotic plaques (Figure 1) after standardized quality control, we applied unbiased clustering on 75,156 cells, identifying 11 cell populations (Figure 2A, Figures S1 and S2C). Cell types were designated to each cluster based on the differential expression of established markers (Figure S2B) within each cluster, representing a mix of cells of carotid and coronary arteries (Figure S2D). The annotation of scATAC-seq populations was transferred from scRNA-seq data, aligning well with the native clustering boundaries of scATAC-seq and preserving the majority of the originally defined scRNA-seq populations (Figure S3A,C). Motifs enrichment of cell type-specific TFs confirmed the annotation between cell types (Figure S3B).

In atherosclerotic plaques, the proportion of immune cells, particularly macrophages and T cells, increased (Figure S3D). The projection of scRNA-seq-derived cell type labels onto spatial RNA-seq predicted the spatial distribution of corresponding cell types in plaques. Macrophages and T cells with a minority of myofibroblasts and ECs were predicted to locate in the plaque core area, while SMCs, myofibroblasts, macrophages, T cells, and few ECs were predicted to reside in fibrous cap area (Figure S3E,F), indicating diverse contribution of cell types to plaque formation.

### 3.2. Foamy Macrophage Identification and Transcriptional Signaling in Human Atherosclerotic Plaques

To comprehensively characterize foamy macrophages, we surveyed feature genes annotating plaque foamy macrophages from public sequencing studies (Figure 2B) [5,8,14,15,16]. The genes, differentially expressed between foam and non-foam cells by RNA sequencing, were associated with lipid formation, transportation, etc. (Figure S4). We hence devised a gene expression score [Foam_score] to quantify the foamy degree for macrophages (Figure 2C), thereby distinguishing the Foam cluster from the Non-Foam cluster (Figure 2D). Spatial mapping of identified foamy macrophages illustrated their confinement to the plaque core and fibrous cap areas, reinforcing the accuracy of our classification (Figure 2E). A heatmap of DEGs showed that foam and non-foam cells were distinct populations (Figure S5A). The feature plot of classic foam cell marker *TREM2* showed a similar distribution between [Foam_score] and *TREM2* expression (Figure S5B). The identified foam cells in mature plaques almost did not overlap with *CD209*^+^*LYVE1*^+^ tissue-resident macrophages, nor with IL1B^+^ inflammatory macrophages (Figure S5B), which is consistent with previous reports on macrophage diversity in atherosclerotic vessels [9]. Compared with non-foamy macrophages, foam macrophages were enriched in cholesterol metabolism and the PPAR signaling pathway (Figure 2F). Notably, high expression of *SPP1* (osteopontin) and enrichment in the osteoclast differentiation pathway suggested a vital role of foam macrophages in regulating plaque calcification (Figure 2F and Figure S5C). Our findings also implicated ferroptosis under oxidative stress as a prevalent mode of programmed cell death in atherosclerotic foam cells (Figure 2G and Figure S5D).

For further identification, we transferred foam and non-foam labels of scRNA-seq data to scATAC-seq data (Figure 2H). The integrated scRNA-seq and scATAC-seq data showed foamy macrophages accounted for 7.36% of macrophages from non-diseased arteries and 33.12% of macrophages from atherosclerotic arteries (Figure 2I), suggesting their early emergence and functional involvement in plaque formation. Compared with non-foamy macrophages, ETS family transcription factors (e.g., *SPIB*, *EHF*, *ELF*, *GABPA*, *SPIC*) motifs were significantly enriched in common foam macrophages (Figure 2J). In line with the scRNA-seq, *GABPA* is required to control cellular and systemic cholesterol homeostasis as well as early vascular lesion formation [17]. *SPIB* promotes macrophage recruitment [18], while *SPIC* retrains inflammatory responses and iron metabolism in macrophages [19]. The above data are consistent with prior findings that foam macrophages were characterized by low expression of inflammatory-response genes [5,15].

### 3.3. Profiling of Foamy Macrophages in Atherosclerotic Plaques

**Figure 3:**
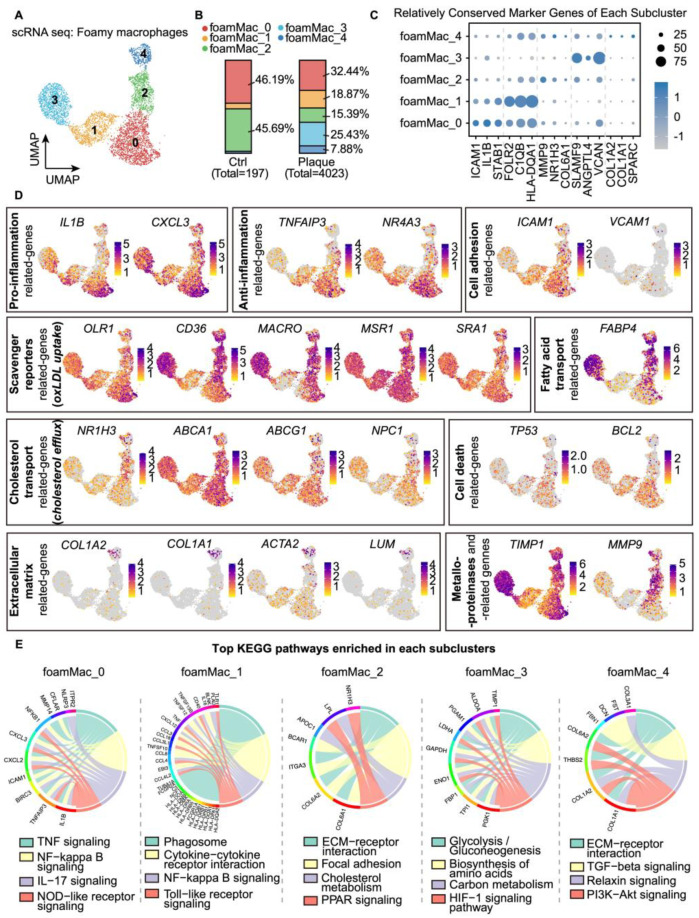
Subclustering of foamy macrophages revealed 5 distinct subsets. (**A**) UMAP embedding of 5 foamy macrophage subsets (4220 cells). (**B**) Bar plot showing the distribution of foamy macrophage subsets across control and plaque groups. (**C**) Dot plot showing expression of top marker genes in foamy macrophage subsets. (**D**) UMAP embeddings of canonical marker genes related to different functions. Normalized gene expression is indicated by color. (**E**) Top KEGG pathways associated with each subset of foamy macrophages.

To provide a more nuanced characterization of foamy macrophages, the foamy macrophage population was further isolated and re-clustered, revealing 5 main subclasses (FoamMac_0 to FoamMac_4) (Figure 3A,C and Figure S6A,B). Among these, FoamMac_0 and FoamMac_2 were posited as the initial foamy macrophages, predominantly comprising foam cells in the early stage of atherosclerosis (group ‘Control’) (Figure 3B). FoamMac_1 and FoamMac_3 represented more advanced and mature foamy macrophages, with their proportions obviously increasing after plaque formation (Figure 3B). Additionally, a small subclass, FoamMac_4, also emerged in atherosclerotic plaques.

FoamMac_0 exerted a relatively inflammatory gene expression profile (e.g., *IL1B*, *CXCL3*, *TNFAIP3*, *NR4A3*) and showed enrichment for pro- and anti-inflammatory responses (Figure 3D,E and Figure S6D). Cells in FoamMac_0 also exhibited heightened expression of *ICAM1* and *VCAM1*, both integral for the recruitment of circulating monocytes/macrophages to the atherosclerotic lesion site [20]. Additionally, *OLR1* (lectin-like oxLDL receptor 1, LOX-1), reported to uptake oxLDL and increase lipid accumulation in the early-stage atherosclerotic plaque [21,22], was upregulated in FoamMac_0. Collectively, these findings suggested that FoamMac_0 might be the initial foamy macrophages, possibly termed “Pro-foam macrophages”, which were likely central to the initiation of foam cell formation in atherogenesis.

In contrast to FoamMac_0 or pro-foam macrophages, FoamMac_1 exhibited a similar gene expression profile but lower expression of inflammatory-related genes. Notably, this subclass displayed relatively higher phagocytic gene expression and was enriched in pathways related to endocytosis and phagocytosis pathways (Figure 3D,E and Figure S6D,E). Despite a lower expression of traditional oxLDL receptor genes (e.g., *CD36* and *MACRO*), FoamMac_1 appeared to possibly uptake and accumulate lipids through exaggerated macrophage phagocytosis in a receptor-independent way [23]. This subpopulation is thus referred to as “Phagocytic foam macrophage”.

FoamMac_3 demonstrated higher expression of genes related to cholesterol uptake (e.g., *CD36* and *MACRO*), storage (e.g., *PLIN2*), and fatty acid transport (e.g., *FABP4* and *FABP5*) (Figure 3D and Figure S6C), indicating an advanced stage of lipid influx and accumulation. Furthermore, FoamMac_3 was enriched for the HIF-1 signaling pathway (Figure 3E), a pathway typically associated with the hypoxic microenvironments prevalent in atherosclerotic lesions. Within macrophages, HIF-1 α activation, triggered by exposure to oxLDL or necrotic debris, not only accelerated foam cell formation but also exacerbated cellular apoptosis [4,24].

FoamMac_2 and FoamMac_4 poorly expressed inflammatory-related genes but exerted an ECM-related gene expression profile (Figure 3D,E). While expressing receptor genes of oxLDL uptake (e.g., *CD36*, *MSR1*, and *MACRO*), these subsets had higher expression of *NR1H3* (LXRα) with enrichment of the PPAR- and LXR- signaling pathways. These pathways are known to enhance cholesterol efflux and regulation of lipid metabolism [25,26]. Interestingly, FoamMac_4 especially expressed *COL1A2* and *COL1A1*, common markers of fibroblasts and enriched for ECM organization (Figure 3D and Figure S6E). This suggested that FoamMac_4, termed a “synthetic foam macrophage”, might specialize in secreting and assembling collagen fibers to form a stabilizing fibrous cap.

### 3.4. Cell Fate Analyses of Foamy Macrophages in Atherosclerotic Plaques

**Figure 4:**
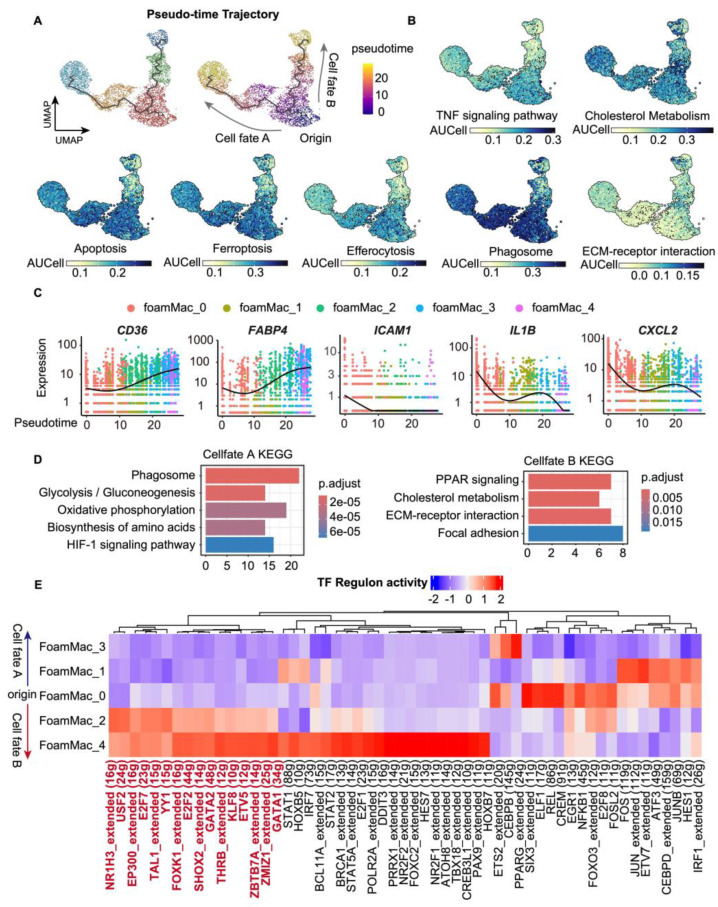
Pseudotime and TF regulon activity analyses for the foamy macrophage subsets. (**A**) UMAP embeddings showing the pseudotime trajectory calculated with Monocle3 (4220 cells). The numbered circle depicts the trajectory root defined as the pro-foam subset. (**B**) UMAP embeddings showing the activity of each pathway calculated by AUCell in the foamy macrophage subsets. (**C**) Expression levels of genes along pseudotime. (**D**) Top KEGG pathways associated with pseudotime cell fate A or cell fate B. (**E**) TF regulon activity prediction of foamy macrophage subsets. *p*. adj, adjusted *p*-value by Benjamini–Hochberg procedure.

To examine potential relationships among the identified foamy macrophage subclasses, we employed trajectory learning and pseudotime defining in the UMAP. Trajectory analysis predicted that FoamMac_0, the initial pro-foam macrophages, have two developmental trajectories: one progressing to FoamMac_1 and ultimately maturing into FoamMac_3 [cell fate A], and the other leading to FoamMac_2 and FoamMac_4 [cell fate B] (Figure 4A). The origin of cell fates FoamMac_0 showed an enrichment of the TNF signaling pathway, which was related to inflammation and reported to promote a shift toward a proatherogenic lipid profile [27]. Along both developmental trajectories, the gene sets related to the TNF signaling pathway were downregulated, concurrent with the upregulation of gene sets involved in cholesterol metabolism (Figure 4B). This shift in gene expression profiles signified foamy macrophage maturation and atherosclerosis progression along pseudotemporal trajectories (Figure 4C), supporting our setting of the starting point for the pseudotime analysis.

Cell fate A and cell fate B likely represented different foamy macrophage outcomes. Cell fate A was notably characterized by the enrichment of programmed cell death pathways (e.g., cell apoptosis, ferroptosis, and necroptosis) with efferocytosis gene sets upregulated in the middle (FoamMac_1) and downregulated at the end (FoamMac_3) (Figure 4B,D). The increasing dead cells and efferocytosis impairment at the termination of cell fate A were supposed to enlarge the necrotic cores in the atherosclerotic plaques, reduce the plaque stability, and further result in secondary necrosis [6]. In contrast, cell fate B barely expressed genes related to cell death but was enriched in ECM-related pathways (Figure 4B,D). Furthermore, the upregulation of cholesterol efflux, ECM organization, and contractile proteins (e.g., *ACTA2*) embedding at the termination of cell fate B implied a contribution to the formation, reinforcement, and thickening of the fibrous cap [28]. This trajectory analysis highlighted that, at a transcriptional level, the foamy macrophage population appeared to make a choice between life and death, further influencing plaque progression.

To predict transcription factors driving the gene expression profile of various foamy macrophage subclasses in cell fates, we performed transcription factor enrichment analysis (Figure 4E). FoamMac_0 and FoamMac_1 showed higher regulon activities of *AP-1*(JUN/JUNB-FOS), known to mediate inflammatory responses [29]. *NR1H3*, a key regulator of macrophage function involved in lipid homeostasis [30], exhibited increased regulon activity in FoamMac_2 and FoamMac_4 [cell fate B]. This finding underscored the importance of *NR1H3* in guiding foamy macrophages along a ‘living’ developmental route and toward a more synthetic phenotype. Interestingly, the regulon activities of some TFs related to estrogen receptor (ESR)-mediated signaling (e.g., *YY1*, *USF2*) were specifically increased in subclasses along cell fate B. As estrogen is suggested to contribute to atherosclerotic plaque stabilization by inhibiting TNF-*α* and activated NFκB-dependent inflammation [31], this observation hinted at a link between the atherosclerotic-protective action of ESR signaling [32] and the developmental choice of foamy macrophages. Interestingly, the regulon activity of *BRCA1*, a tumor-suppressor gene and potential regulator of heart failure [33], was also increased in FoamMac_2 and FoamMac_4 along cell fate B.

In the scATAC-seq data, subclass labels transferred from scRNA-seq to scATAC-seq subclasses showed similar marker gene expression (Figure S7A). The differential open chromatin TF motifs among transferred labels were still detected and partly in line with scRNA-seq data (Figure S7B).

### 3.5. Immunostaining of the Foamy Macrophage Subsets in Human Atherosclerosis

**Figure 5:**
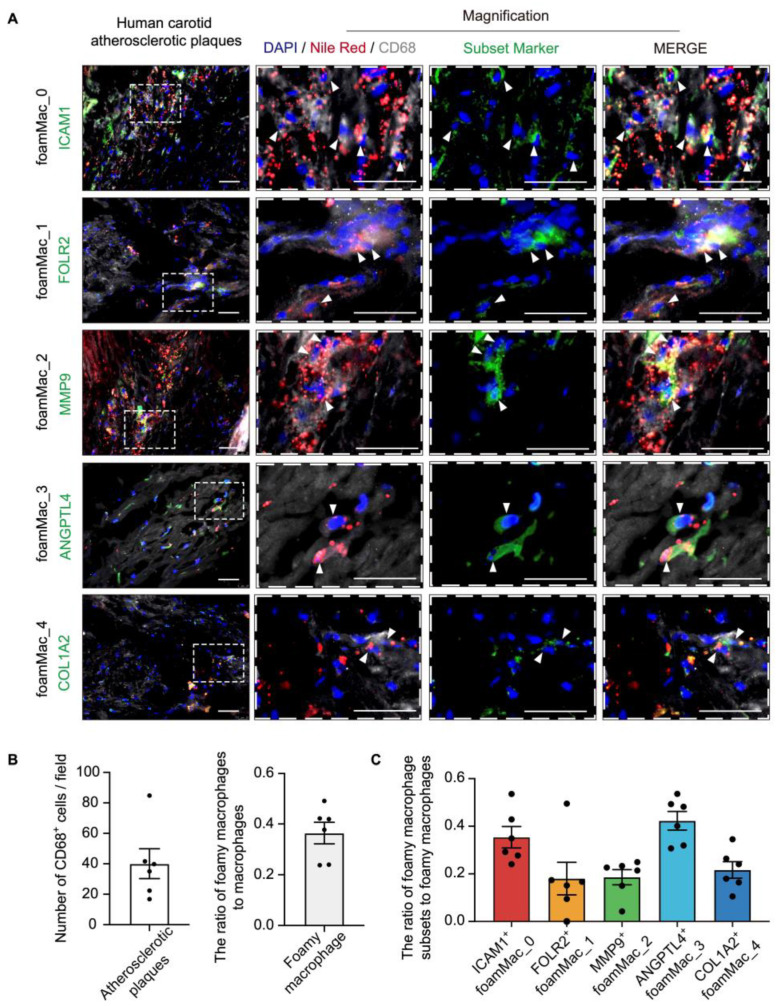
Immunostaining of the foamy macrophage subsets in human atherosclerosis. (**A**) Immunostaining for Nile Red, CD68, foamy macrophage subset markers (ICAM1, FOLR2, MMP9, ANGPTL4, and COL1A2) and DAPI on human carotid atherosclerotic plaques sections. Arrows indicate Nile Red^+^ CD68^+^ subset marker^+^ cells. (**B**,**C**) Quantification of foamy macrophages in macrophages (**B**) and each foamy macrophage subset in foamy macrophages (**C**). Data are mean ± SEM; n = 6, biological replicates. Scale bars: 50 μm.

To validate the existence of these foamy macrophage subsets, we conducted immunostaining in atherosclerotic human carotid plaques. Foamy macrophages were defined as lipid-laden macrophages [3]. Therefore, we stained foamy macrophages for lipid droplet stain Nile Red and CD68 and detected that 36.43 ± 4.24% of CD68^+^ macrophages were foamy macrophages (Figure 5A,B). Moreover, foamy macrophage subsets were further signed by specific marker genes (Figure 3C). Immunostaining for Nile Red, CD68, and foamy macrophage subset markers (FoamMac_0 [ICAM1^+^], FoamMac_1 [FOLR2^+^], FoamMac_2 [MMP9^+^], FoamMac_3 [ANGPTL4^+^], and FoamMac_4 [COL1A2^+^]) confirmed the existence and location of each foamy macrophage subset in human carotid plaques (Figure 5A). The ratio of each macrophage subset to foamy macrophages was in line with the distribution of their respective single-cell subsets (Figure 5C).

### 3.6. Intercellular Communication of Foamy Macrophage Subpopulations Drives Inflammatory Interactions Within the Lesion

**Figure 6:**
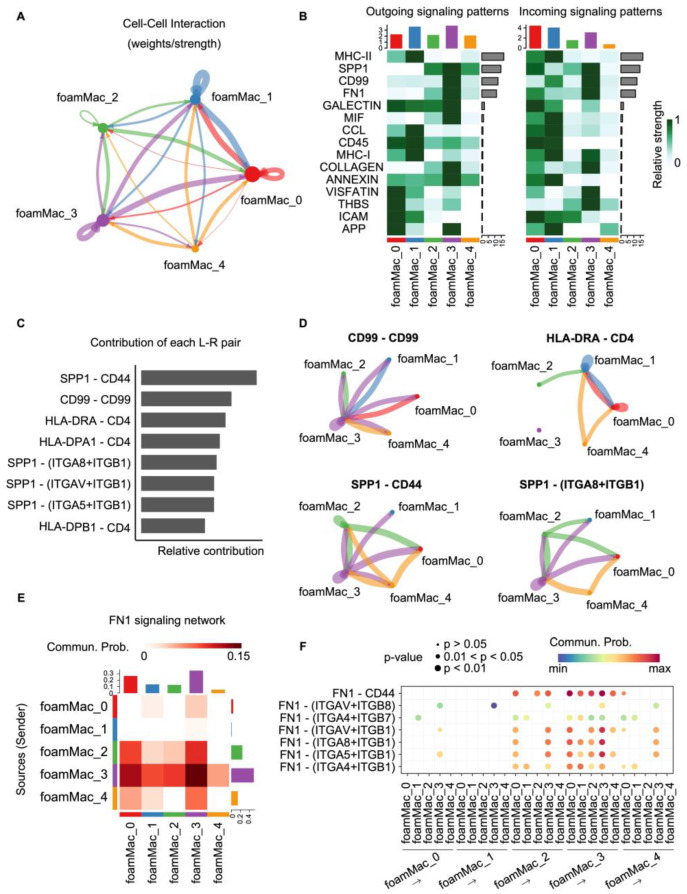
Summary of cell crosstalk within the foamy macrophage subsets in human atherosclerosis. (**A**) Circle plot depicting aggregated cell–cell interaction network within foamy macrophage subsets. (**B**) Heatmap showing the top signal pathways of cell–cell communication within foamy macrophage subsets. (**C**) Bar plot showing the contribution of ligand–receptor pairs in cell–cell communication within foamy macrophage subsets. (**D**) Circle plots depicting ligand–receptor interactions within foamy macrophage subsets for CD99-CD99, SPP1-CD44, SPP1-(ITGA8+ITGB1), and HLA-DRA-CD4. (**E**) Heatmap depicting cell–cell interactions within foamy macrophage subsets for FN1 signaling. (**F**) Dot plot of ligand–receptor interactions for FN1 signaling. L-R, ligand–receptor.

We next explored the potential intercellular communication within the atherosclerotic lesion by examining ligand–receptor interactions among the various foamy macrophage subpopulations. Foam populations showed robust potential interactions with each other, with FoamMac_3 and FoamMac_1 populations displaying particularly strong interactions with other populations (Figure 6A).

Subsequently, we identified several potentially prominent signaling axes, including FoamMac_3 sending Osteopontin (SPP1), FoamMac_1 sending MHC-II, FoamMac_3 sending fibronectin 1 (FN1) and FoamMac_3 sending CD99 (Figure 6B). Ligand–receptor contribution was further calculated (Figure 6C). SPP1/CD44 interactions, known to play an immunosuppressive role [34], were primarily from more developed foamy macrophage subpopulations (e.g., FoamMac_3 and FoamMac_4) (Figure 6D). CD44, besides facilitating immune evasion, also promoted foam cell adhesion and macrophage recruitment to lesions, underscoring its significance in atherosclerotic progression [35]. We also observed potential interactions from SPP1 on relatively mature foamy macrophage subpopulations (e.g., FoamMac_3 and FoamMac_4) to integrin α8β1 (ITGA8+ITGB1) on most foamy subpopulations (Figure 6D). CD99-CD99 interactions were possibly mainly from FoamMac_3 and targeted every foamy macrophage population (Figure 6D). This interaction is potentially associated with cell migration and adhesion, especially transendothelial leukocyte migration [35]. Moreover, HLA-DRA-CD4 were predicted to interact between foamy macrophage subpopulations except FoamMac_3, leading to mutual activation and facilitating the antigen presentation process [36]. For the FN1 signal, which mainly included FN–integrin pairs, the FoamMac_3 subpopulation seemed to be the main sender and strongly interacted with other foam populations, especially FoamMac_0 (Figure 6E,F). The FN signaling shows proinflammatory properties by enhancing integrin signaling, which is reported to promote atherosclerotic plaque formation [37,38]. Notably, we found that FoamMac_3 strongly contributed to FN1 signaling, while the contribution of FoamMac_2 or FoamMac_4 was relatively weak. This may be because the great contributions of FN–integrin pairs may mask the contributions of some other pairs. This interaction enhances inflammatory pathways to increase the recruitment of inflammatory cells at atheroprone sites and further improves atherosclerosis [37,39], implying the recruitment role of mature foamy macrophages to initial foamy macrophages.

### 3.7. Mendelian Randomization Analyses Revealed Possible Causal Relationship Between Cell Trajectory Genes and Atherosclerotic Diseases

**Figure 7:**
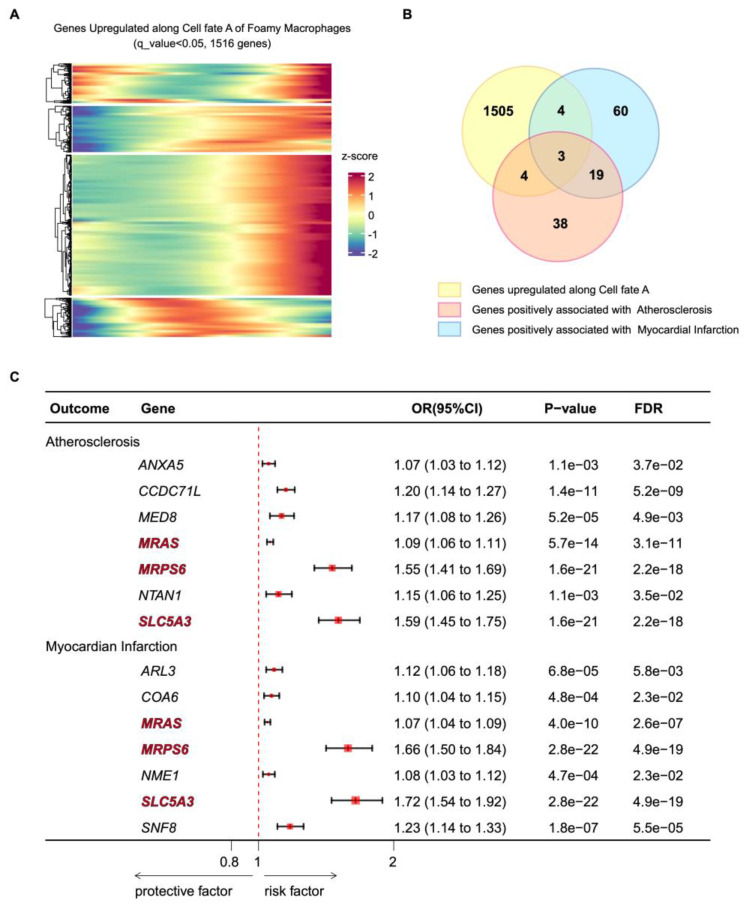
Mendelian randomization analyses revealed a possible causal relationship between cell trajectory genes and atherosclerotic diseases. (**A**) Heatmap showing the foamy macrophage differentially expressed genes upregulated across pseudotime cell fate A (q value < 0.05). (**B**) Venn plot showing the overlapping genes among upregulated genes across pseudotime cell fate A (q value < 0.05), atherosclerosis-associated genes (FDR < 0.05), and myocardial infarction-associated genes (FDR < 0.05). (**C**) Forest plot of the overlapping genes in (**B**). FDR, false discovery rate. OR, odds ratio.

The above data demonstrates the association between key genes and foamy macrophage fate. Using trajectory analysis of foamy macrophage subclasses, we observed a set of genes upregulated along the cell fate A toward programmed cell death (Figure 7A). This upregulation was expected to enlarge the necrotic cores in atherosclerotic plaques and thereby accelerate plaque rupture. Next, we conducted Mendelian randomization analyses to examine the causal relationship between specific gene expression in artery tissue and the risk of atherosclerosis or MI. The results are presented in Supplementary Table S2. In total, 64 genes were significantly positively associated with atherosclerosis, and 86 genes were significantly positively associated with myocardial infarction at a nominally significant level (FDR < 0.05). After mapping those genes into the programmed cell death gene set, 7 out of 1516 programmed cell death genes were associated with atherosclerosis risk, and 7 genes were associated with MI risk (Figure 7B). In genetically predicted levels, higher expression of *ANXA5*, *CCDC71L*, *MED8*, *MRAS*, *MRPS6*, *NTAN1*, and *SLA5A3* was positively associated with atherosclerosis risk. Higher expression of *ARL3*, *COA6*, *MRAS*, *MRPS6*, *NME1*, *SLC5A3*, and *SNF8* was associated with MI risk (Figure 7C). Notably, *MRAS*, *MRPS6*, and *SLC5A3* were specifically associated with both atherosclerosis and MI risk. These genes, emerging as candidate targets for both conditions, also mapped to foamy macrophages in the context of programmed cell death, offering new avenues for mechanistic exploration.

## 4. Discussion

Here, we combine integrated single-cell analyses, immunostaining of human samples, and Mendelian randomization analyses, revealing a novel high-resolution cellular landscape of foamy macrophages and potential key regulatory genes in human atherosclerotic plaques. A more comprehensive and nuanced classification of human foamy macrophage subclasses with specific trajectories toward distinct cellular destinies is presented. Potential biological mechanisms under the cell fate choice of foamy macrophages are further investigated. Summarily, our study provides a detailed characterization of diverse foam cell subpopulations, offering significant insights into the mechanisms of atherosclerosis.

In recent years, single-cell technologies have advanced our knowledge of atherosclerosis remarkably, depicting the immune and non-immune cell landscape in both murine and human atherosclerotic lesions. Previous research identified *TREM2*^hi^ macrophages in atherosclerotic mice, which had specialized functions in lipid metabolism and catabolism [12]. Another distinct cluster presenting a foam cell transcriptional signature (e.g., *APOC1*, *APOE*, *PLIN2*) was identified from human plaque macrophages [10]. Recent research also discovered distinct macrophage subpopulations characterized by a proliferative gene signature or an SMC-specific gene signature [13]. Research has provided insights into the distinct gene expression profiling of foamy macrophages compared with other non-foamy macrophages. Despite these advances, existing studies have not fully dissected the heterogeneity and potential cell fate within foamy macrophages in atherosclerotic plaques, often due to the scarcity of foam cells in individual studies. Here, we integrate single-cell analyses, including scRNA-seq, scATAC-seq, and spatial sequencing, to reveal a novel cellular landscape of foamy macrophages consisting of five sub-populations.

Establishing a flow cytometry-based method of lipid staining, the main component of lipid-laden foam cells was foamy macrophages (70~80%) [5]. Based on the reported gene expression profile of foamy macrophages, we identified the foamy macrophages by scoring lipid-processing genes. Our approach, which encompassed a comprehensive gene expression profile, inferred functional characteristics, and spatial context, was consistent with previous research and bolstered the validity of our foam cell identification. However, there are still some limitations. As reported, the relative gold standard for identifying foam cells is performing BODIPY staining [40]. While we used DEGs defined by BODIPY staining to identify foam cells, defining foam cells based on RNA expression may underrepresent them, as some transcripts are weakly expressed and shared across similar populations. Nonetheless, the functions associated with these genes are related to foam cell formation or maturation, such as lipid uptake, transport, and storage. Immunostaining of foamy macrophage subsets for lipid droplet stain Nile Red also confirmed the validity of our foamy macrophage identification.

One substantial result from our analyses was the heterogeneity and predicted binary cell fates of human foamy macrophages. While FoamMac_0 seemed to be pro-foam macrophages, cell fate A (FoamMac_1 and FoamMac_3) and cell fate B (FoamMac_2 and FoamMac_4) represented different outcomes: death or survival. Cell fate A (FoamMac_1 and FoamMac_3) showed an increasing expression of lipid uptake and storage genes (e.g., *CD36*, *PLIN2*), as well as the enrichment of apoptosis and other programmed death pathways. These findings aligned with the pathophysiological sequence where macrophage lipid overload matured foam cells and instigated apoptosis, exacerbating necrotic core expansion [3]. FoamMac_1, positioned as an intermediary in the trajectory toward cell death, was significantly characterized by efferocytosis, a crucial process mitigating inflammation and curbing excessive cell death [41]. Conversely, cell fate B showed relatively higher cholesterol efflux in FoamMac_2 and ECM organization in FoamMac_4, potentially contributing to plaque stabilization. Interestingly, we found that the regulon activities of some TFs related to estrogen receptor signaling were increased along cell fate B, which suggested that the atheroprotective effect of estrogen may be related to the cholesterol efflux and matrix synthesis of foamy macrophages.

Previous studies have described an individual subset of foamy macrophages and their role in protecting from or promoting atherosclerosis [16,42,43]. *TREM2^hi^* myeloid-derived macrophage is regarded as one of the main subtypes in plaque macrophages, identified as foamy lipid-laden macrophages [12,15]. TREM2 was reported to protect from atherosclerosis by limiting necrotic core formation, which is essential for macrophage efferocytosis [42]. However, TREM2 might also foster cholesterol uptake and foam cell genesis, with myeloid-specific *Trem2* deletion attenuating plaque progression [16,42]. The controversial role of TREM2 or TREM2^hi^ macrophages may be due to the heterogeneity of foamy macrophages. Our findings echoed this complexity, with *TREM2* expression peaking in FoamMac_1, FoamMac_2, and FoamMac_4 but reduced in FoamMac_0 and FoamMac_3, implicating its involvement in both foam cell biogenesis and efferocytosis regulation in FoamMac_1. Previous research revealed the existence of *PLIN2*^hi^/*TREM1*^hi^ macrophages with signatures of lipid handling, inflammation, and apoptosis [44]. In line, our study showed that *TREM1* and *PLIN2* simultaneously and significantly increased in the terminate state of cell fate A to programmed death (FoamMac_3). Consistent with the report of a foamy macrophage cluster expressing the SMC marker *ACTA2* [8], our FoamMac_4 cluster resonated with the above observations. *Slamf9*, used to annotate a subtype of *Trem2*^hi^ macrophages in murine atherosclerosis [15], was parallelly expressed in FoamMac_3 in human plaques. By integrating single-cell sequencing of human atherosclerosis, we present a more comprehensive and nuanced classification of human foamy macrophage subclasses, elucidating specific trajectories toward distinct cellular destinies.

Apart from the putative functions of foamy macrophage subclasses, the differentially enriched regulon activities were calculated to predict potential TF driving the cell fate choice. For instance, the regulator *NR1H3*, as well as some ESR-related TFs, were specially activated along the living cell fate toward synthetic phenotypes (cell fate B). This aligned with transcriptomic changes, suggesting these regulators as potential therapeutic targets for plaque stabilization. Furthermore, we identified the DEGs along the cell trajectory toward programmed cell death. By MR analysis, these DEGs (e.g., *MRAS*, *MRPS6*, and *SLC5A3*) were linked to causal effects with adverse outcomes in atherosclerosis and MI. Interestingly, our analysis revealed distinct expression profiles of scavenger receptor genes across foamy macrophage subclasses. Scavenger receptor LOX-1(OLR1) potentially functioned in the initial foam cells, as reported in [22], while scavenger receptors CD36 and MACRO were supposed to function in the mature foam cells. Scavenger receptor MSR1 might work through the entire process of foam cell formation. These findings highlighted the dynamic roles of scavenger receptors in the different stages of plaque formation.

Our study has a few limitations. Although the *Harmony* algorithm was applied to solve the biological and technical differences [45], patients represented highly different characteristics (e.g., age, sex, comorbidities such as diabetes, etc.), which are known to influence plaque cell composition [46,47]. Moreover, MR analysis has limitations related to heterogeneity across different vascular tissues and human populations. Therefore, further confirmation by more cell assays, animal experiments, and clinical data will be necessary.

## 5. Conclusions

In summary, by integrating single-cell libraries, we identified the foamy macrophage subsets in human atherosclerosis at unprecedented resolution. We further re-clustered the foamy macrophages into five unique subsets with distinct potential functions. These foamy macrophage subsets formed a binary differentiation cell trajectory toward two distinct cell outcomes: the programmed death or synthetic state. Through TF regulon analysis, we inferred key drivers steering the developmental trajectory of foamy macrophages. These foam populations have strong potential ligand–receptor interactions among them. MR analysis identified the causal relationship between foamy cell fate A-related genes (*MRAS*, *MRPS6,* and *SLC5A3)* and the incidence of atherosclerosis and myocardial infarction. Our study raised in-depth characterization of the diverse foamy macrophage subpopulations and a set of potentially key regulatory genes in atherosclerotic plaques.

## Data Availability

Public datasets used in this study are described in Figure 1 or Supplementary Table S2. R scripts have been deposited on GitHub and are publicly available as of the date of publication. Other data are available from the corresponding authors upon reasonable request.

## Supplementary Materials

The following supporting information can be downloaded at https://www.mdpi.com/article/10.3390/biom14121606/s1: Figure S1: Public datasets used to build the integrated single-cell analysis for human atherosclerosis; Figure S2: Integration of single-cell RNA sequencing datasets; Figure S3: Integration of single-cell RNA-sequencing, spatial transcriptomic and chromatin accessibility sequencing datasets; Figure S4: The genes used to calculate Foam_Score and their reported function; Figure S5: Characterization of foamy and non-foamy macrophages in human atherosclerotic plaques; Figure S6: Characterization of distinct foamy macrophage subsets in integrated scRNA-seq data; Figure S7: Characterization of foamy macrophage subsets in integrated scATAC-seq data; Table S1: Information of datasets used in the study; Table S2: Detailed information of MR analyses; Table S3: Strobe MR checklist.
