# Supplementary material for: Integrated Single-Cell Analysis Revealed Novel Subpopulations of Foamy Macrophages in Human Atherosclerotic Plaques
Source: Biomolecules. 2024 Dec 16;14(12):1606. doi: 10.3390/biom14121606 (PMC11675068; doi:10.3390/biom14121606)
Supplement: Supplementary file 1 [file biomolecules-14-01606-s001.zip › Supplementary Figures.pdf]

Datasets used in the study

Technique	Publication	PMID	GEO ID	Data
scRNA seq	Paloschi V et al. (2023)	37797407	GSE247238	Human carotid arteries
	Alsaigh T et al. (2022)	36224302	GSE159677	Human carotid arteries
	Pan H et al. (2020)	32962412	GSE155512	Human carotid arteries
	Yao F et al. (2018)	29871976	GSE112417	Human carotid arteries
	Wirka RC et al. (2019)	31359001	GSE131778	Human coronary arteries
scATAC seq	Turner AW et al. (2022)	35590109	GSE175621	Human coronary arteries
Spatial Transcriptomics	Theofilatos K et al. (2023)	37646165	GSE241346	Human carotid arteries
	Kawai K et al. (2023)	37881937	GSE243179	Human coronary arteries

Supplementary Figure S1

Public datasets used to build the integrated single cell analysis for human atherosclerosis.

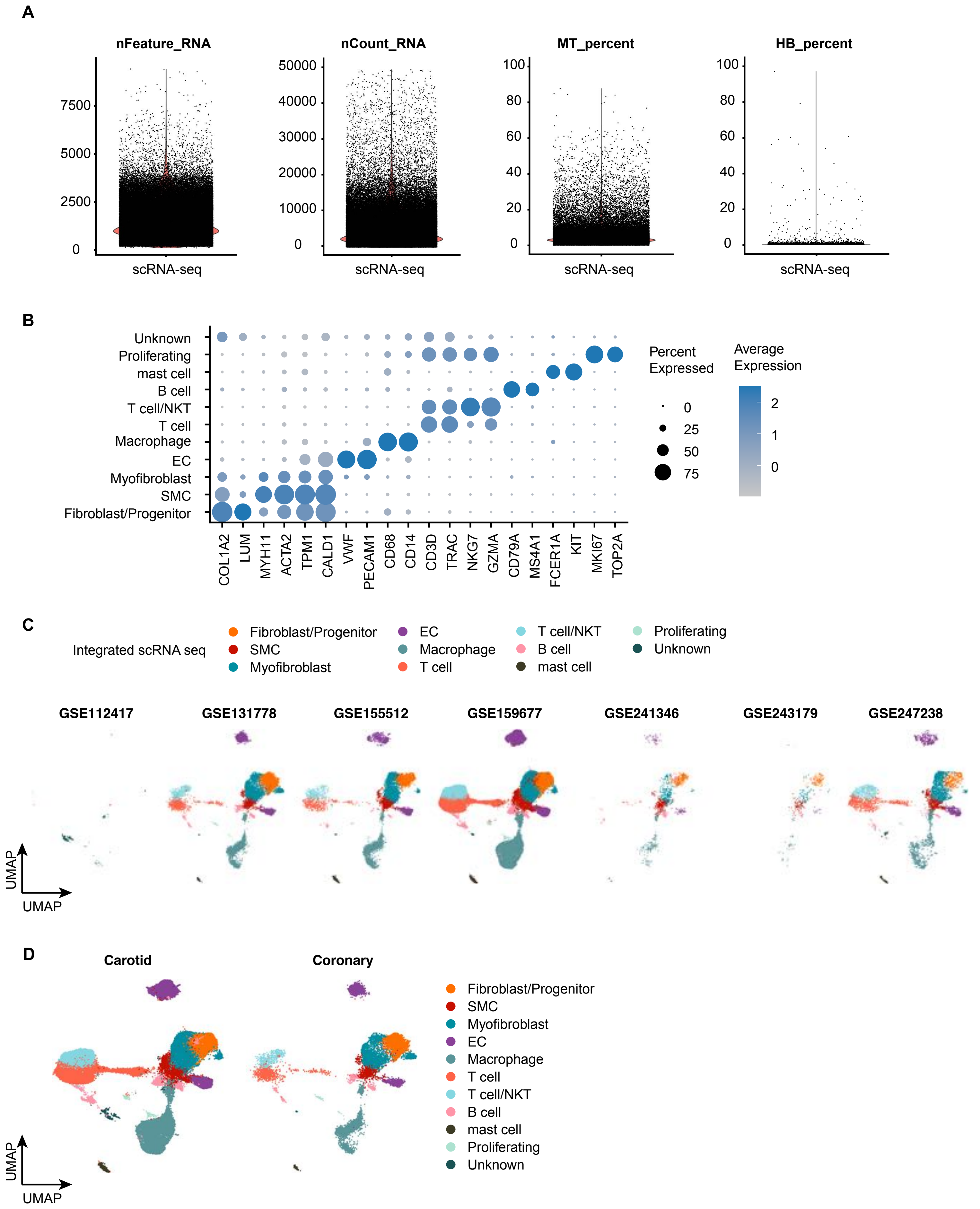

Supplementary Figure S2

(A) Violin plots showing the distribution of the number of unique genes, the number of total molecules, mitochondrial percents and hemoglobin percents.

(B) Dot plot showing expression of canonical cluster-identifying genes.

(C) UMAP representation of integrated scRNA-seq data across reference datasets (75,156 cells).

(D) UMBAP representation of integrated scRNA-seq data across carotid and coronary arteries (75,156 cells).

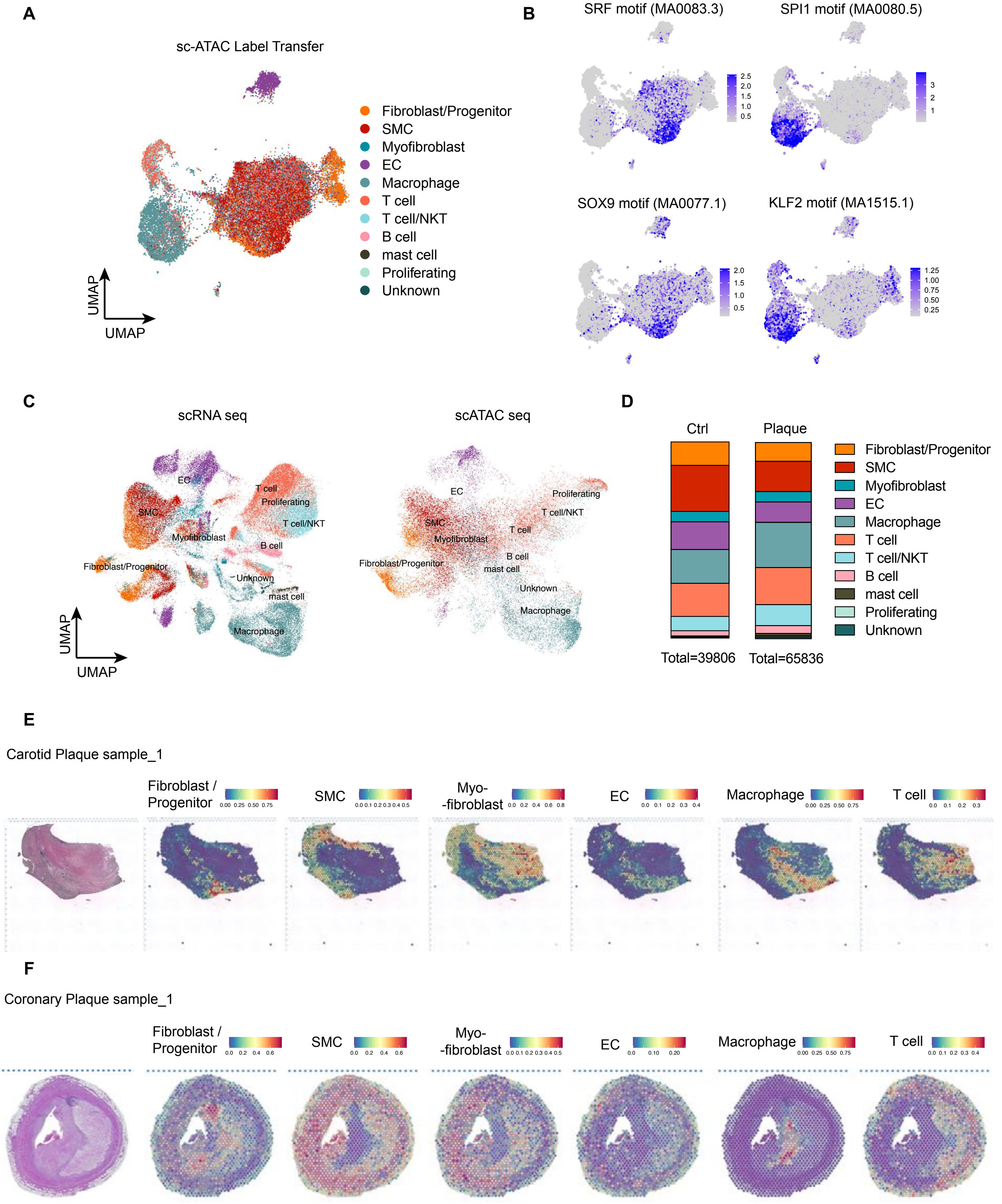

Supplementary Figure S3

(A) UMAP representation of integrated scATAC-seq data from human atherosclerotic plaques with labels transferred from integrated scRNA-seq data (30,445 cells).
(B) UMAP embeddings of transcription factor motifs expression in integrated scATAC-seq data.
(C) Co-embedding UMAP of integrated scRNA-seq and scATAC-seq data (105,601 cells).
(D) Bar plot showing the distribution of cell populations across control and plaque groups.
(E and F) Predicted spatial location of major cell populations in the sections of human carotid (E) and coronary (F) atherosclerotic plaques.

The Genes Used to Calculate Foam_Score and Their Reported Function

Gene Name	Reported Molecular Function	Reference (PMID)
ABCA1	Regulate cellular cholesterol and phospholipid homeostasis	12740222; 11752403
ABCG1	Mediate efflux of cellular cholesterol to HDL	15210959; 16054053
CD36	oxLDL uptake and foam cell formation	11560944; 17442283
TREM2	Surface lipid receptor; regulate foamy macrophage differentiation	38646596; 8695172
LIPA	Cholesterol ester hydrolase	31645127; 26241101
LGALS3	Lipid endocytosis	32149158; 9588889
CD9	Cell adhesion	32346137;
CTSD	LDL uptake and foam cell formation	28078176; 18396408
FABP5	Bind free fatty acids and regulate lipid metabolism and transport; intracellular lipid chaperones	21474828
MERTK	Regulate efferocytosis	28067670; 18451332
NR1H3	Activate cholesterol efflux	24695022; 12193651
NPC1	Intracellular cholesterol trafficking	18483620; 17984379
FABP4	Lipid chaperones	18551191; 30726793
MSR1	Mediate glycated LDL transcytosis	9069289; 9564887
SRA1	Regulate adipocyte differentiation	21152033; 10199399
SCARB1	Mediate selective cholesterol uptake	8560269; 10066776
PLIN2	Lipid droplets formation and stability	16884492; 14707038
MMP9	Plaque development and cellular accumulation	15746435
OLR1	Mediate oxLDL uptake	33176449; 24419805
APOE	Reverse cholesterol transport	15681305; 10882339
NR1H2	Activate cholesterol efflux	24695022; 21493922

Supplementary Figure S4

The table of genes used to calculate Foam_Score and their reported function.

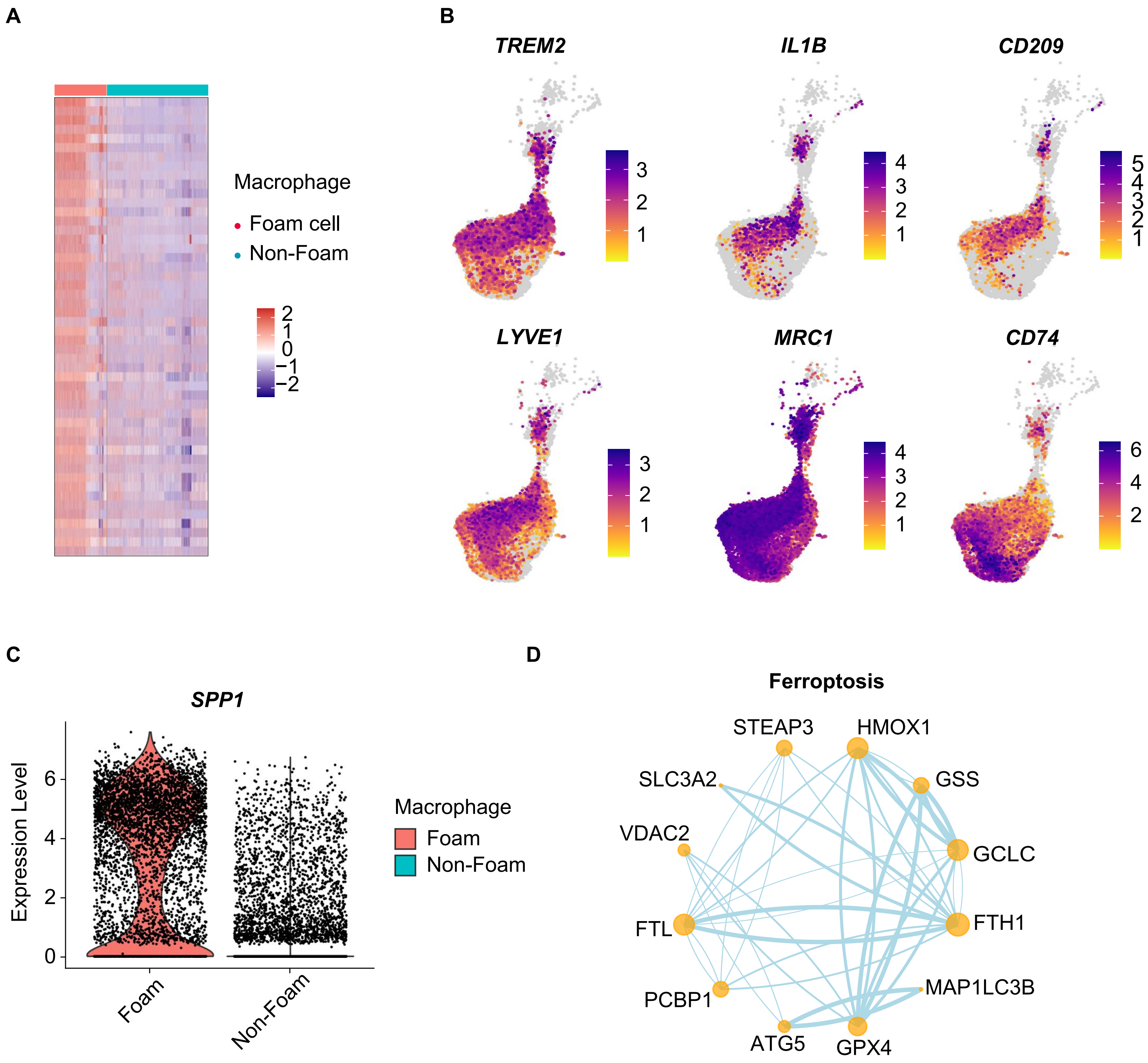

Supplementary Figure S5

(A) Heatmap of top marker genes between 'Foam' and 'Non-Foam' clusters.

(B) Featureplot showing the distribution of marker genes expression in macrophages.

(C) Violin plot showing *SPP1* gene expression in 'Foam' and 'Non-Foam' clusters of macrophages.

(D) Circle plots showing protein-protein interaction of ferroptosis pathway genes associated with 'Foam cell' cluster.

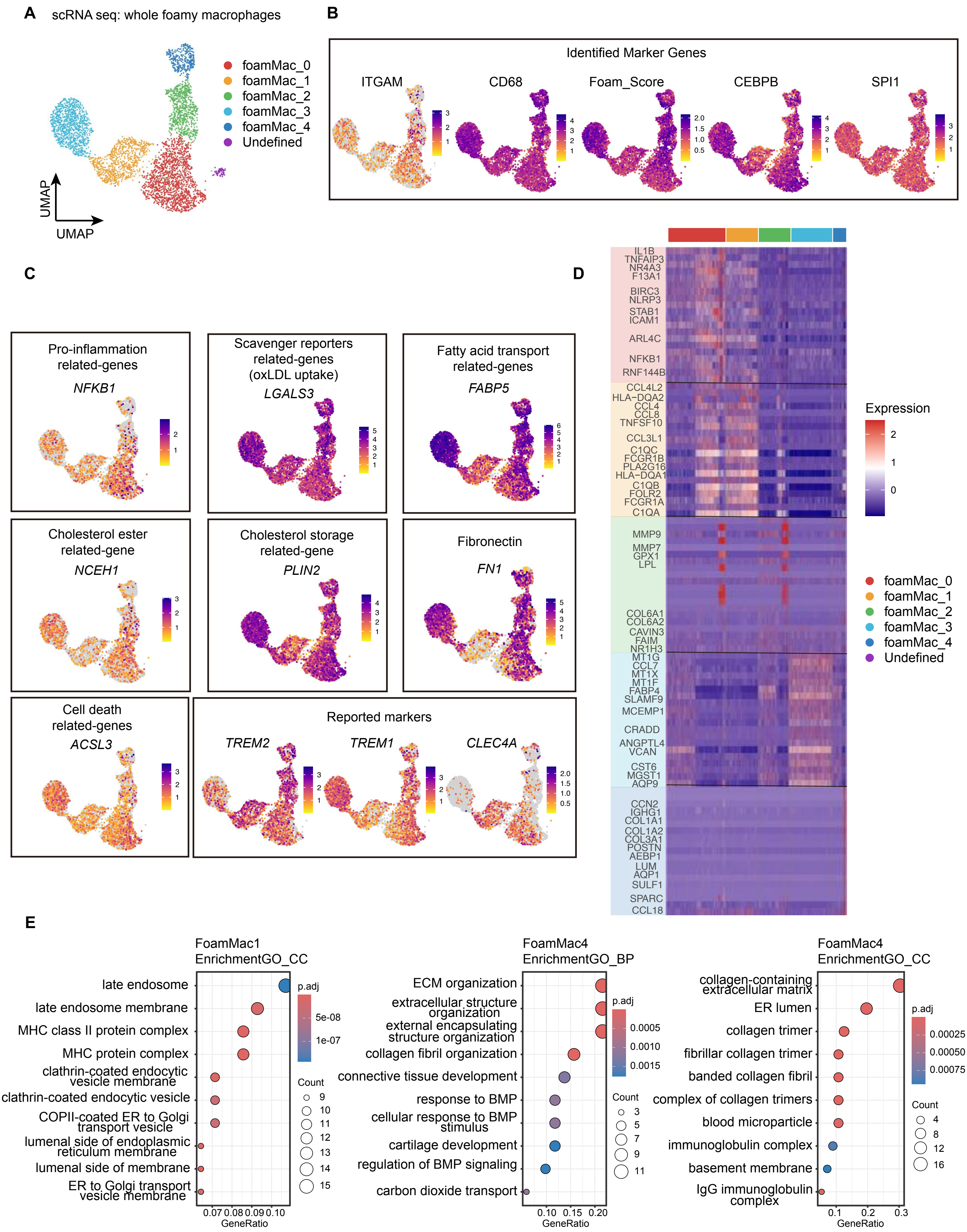

Supplementary Figure S6

(A) UMAP embedding of foamy macrophage subsets (4,282 cells). (B) UMAP embeddings of identified marker genes in foamy macrophage subsets. (C) UMAP embeddings of canonical marker genes related to different functions or reported marker genes. (D) Heatmap of top differential expression marker genes among foamy macrophage subsets. (E) Top Gene Ontology pathways associated with each subset of foamy macrophages. P. adj, adjusted p value by Benjamini-Hochberg procedure.

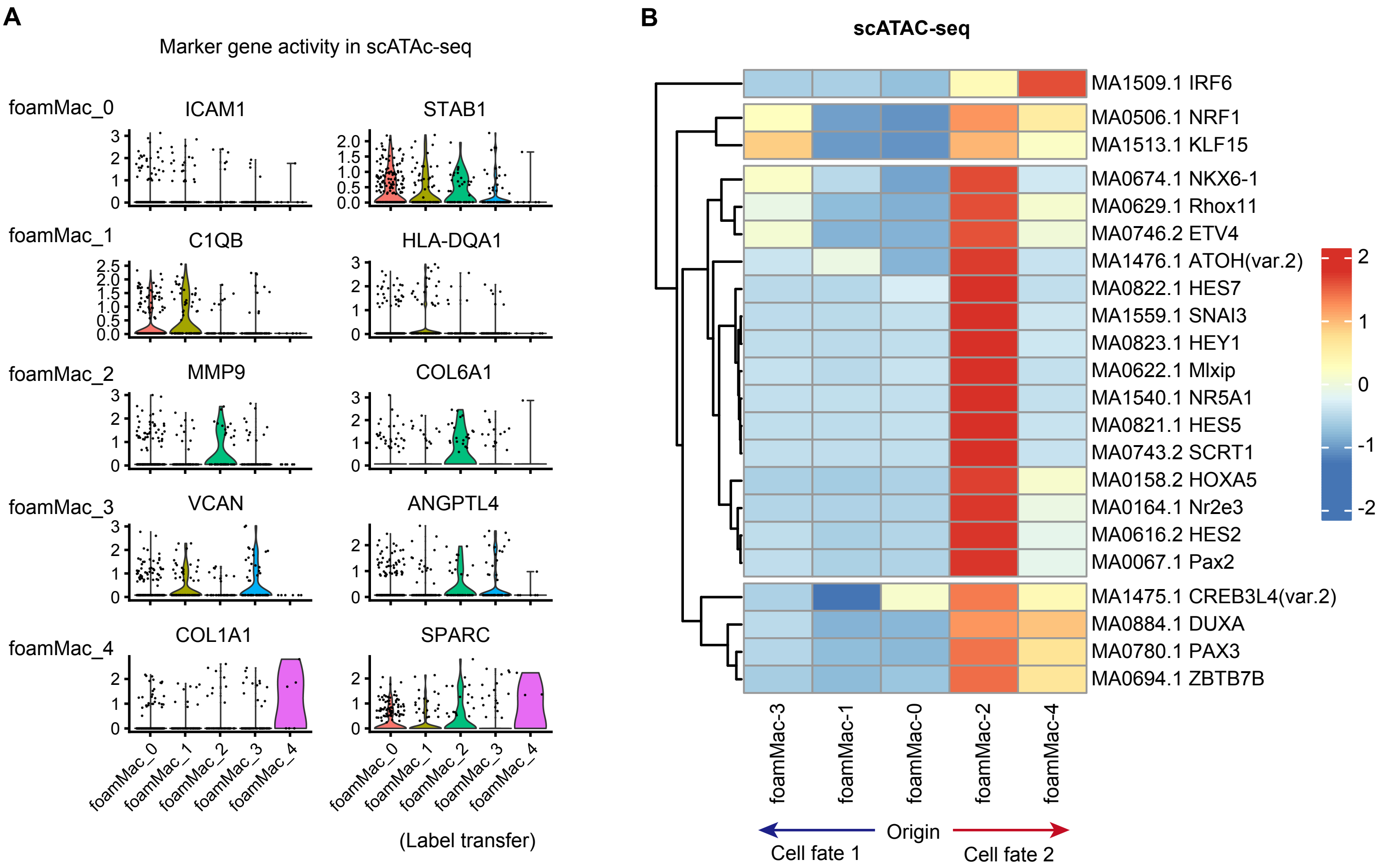

Supplementary Figure S7

(A) Violin plots of foamy macrophage subset marker gene activities in foamy macrophage subsets from integrated scATAC-seq data. The labels were transferred from integrated scRNA-seq data.

(B) Heatmap of differentially expressed transcription factor motifs among foamy macrophage subsets in integrated scATAC-seq data.
